# Investigating the effects of cervical collar design and fit on the biomechanical and biomarker reaction at the skin

**DOI:** 10.2147/MDER.S149419

**Published:** 2018-03-15

**Authors:** Peter R Worsley, Nathan D Stanger, Aran K Horrell, Dan L Bader

**Affiliations:** Clinical Academic Facility, Faculty of Health Sciences, University of Southampton, Southampton, UK

**Keywords:** cervical collar, pressure ulcer, device design, biomechanics, biomarkers

## Abstract

**Background:**

Research has shown that up to 33% of pressure ulcers (PUs) acquired in hospitals result from the application of a medical device. Cervical collars (C-collars) have been implicated in causing PUs, due to the mechanical force they apply to the skin. In order to improve our understanding of collar-related PUs, the present study aimed to assess the biomechanical, biochemical, and microclimate effects of C-collar design and fitting tension.

**Methods:**

A cohort of 15 healthy volunteers was fit with two different C-collars according to the manufacturer guidelines. Two further collar tensions were also defined as loose and tight for each device. Each collar condition was applied for 15 minutes, with a 10 minute refractory period. Measurements at the device–skin interface included interface pressures, inflammatory biomarkers, microclimate, range of cervical motion, and comfort scores.

**Results:**

The interface pressures at each tissue site increased monotonically with greater collar tension (*p*<0.01), irrespective of collar design. Biomarker analysis revealed that inflammatory cytokines (IL-1a) were elevated during collar application, with the highest increase during the tight fit condition, representing over a fourfold increase from unloaded conditions. Regardless of collar tension or type, there was an increase in temperature 1.5°C ±0.8°C compared to baseline values. Range of motion significantly decreased with greater strap tension (*p*<0.05), with an associated increase in discomfort.

**Conclusion:**

The present findings revealed that increasing C-collar tensions caused elevated contact pressures at the device–skin interface, with a corresponding inflammatory response at the skin. These peak contact pressures were highest at the occiput, corresponding with reported PU locations. Devices should be designed to uniformly distribute pressures, and appropriate guidance is needed for their application.

## Introduction

Individuals who have experienced a serious trauma to the head or neck are routinely immobilized with a cervical collar (C-collar) until potential fractures or ligamentous injury are examined.^1^ C-collars are also used in the nonacute setting to manage cervical injury by providing biomechanical support to the head and neck during musculoskeletal rehabilitation.^2^ In order to limit mobility, collars are fixed securely to the neck via strapping and height adjustment, creating points of increased pressure often associated with shear forces at the skin–device interface. These pressure and shear forces can cause a high risk of skin breakdown and the development of pressure ulcers (PUs), also termed pressure injury.^3^ Several devices which are attached to the body have been implicated in PUs, resulting in the adoption of a new term “medical device-related pressure ulcers” (MDRPUs) in international guidelines.^4^ Recent evidence has revealed MDPRUs account for approximately a third of all hospital-acquired PUs, and their presence increases the risk of PUs developing by 2.4 fold.^5^

Common sites of skin breakdown specifically associated with C-collars include the occiput, mandible, ears, chin, laryngeal prominence, shoulders, and sternum.^6^ Many trauma patients, particularly those who are critically ill, have increased susceptibility to PUs^7^ due to inherent factors such as reduced consciousness,^8^ response to noxious stimuli,^9^ and reduced intrinsic tolerance to pressure. In addition, specific subgroups of patients such as the spinal cord injured who are stabilized with C-collars have been reported to exhibit a relatively high incidence of occiput MDRPUs.^10^ Prevalence values for collar-related tissue breakdown have been reported to range from 7%–38%^3^ and documented to be as high as 55% when worn for greater than 5 days.^11^ However, much of the existing literature with respect to C-collars involves observational studies concerned with PU incidence.^12^

The few experimental studies comparing different collars have focused primarily on interface pressure measurements.^13^–^15^ However, the variability in the individual tissue tolerance to pressure magnitude and duration limit the prognostic value of these measures.^16^ Tissue tolerance can be a factor of intrinsic health status such as immobility, comorbidities, and nutritional status.^17^ Thus, there is a need to monitor the physiological response of the skin during device application. Recent research investigating skin health has identified inflammatory cytokines as suitable biomarkers to monitor the physiological response of skin to pressure and shear.^18^ Subsequently, experimental studies have been conducted to investigate the effects of respiratory mask design and application on the biomechanical and physiological response of the skin tissue.^19^ The present study adopted a similar approach, whereby biomechanical, biochemical, and microclimate responses to different C-collar designs and fitting protocols were assessed. The aim of the study was to establish whether the design and application of C-collars influenced the status of skin health.

## Patients and methods

A randomized crossover design was used in the present study.

### Participants

A convenience sample of able-bodied participants was recruited from the local university population using poster advertisement. Participants were included if they were aged between 18–65 years. Exclusion criteria included illness, reduced tolerance to supine lying, or conditions affecting either the cervical spine or skin at the site of C-collar application eg, osteoarthritis or musculoskeletal injury. Additionally, individuals with a history of cardiovascular insufficiency or systemic inflammatory conditions were excluded to avoid the risks of tissue ischemia and perturbation of the biomarkers, respectively. Written informed consent was obtained from each participant prior to testing including publication of images.

Ethics approval was granted from the University of Southampton Ethics Committee (FOHS-ERGO-18511).

A sample of 15 healthy volunteers, 9 males and 6 females, were recruited. The participants had a mean age of 24 years (range 24–31), mean height of 1.72±0.08 m, and mean weight of 67±12 kg, with a corresponding body mass index (BMI) of 23±3 kg/m^2^.

### Test equipment

**Figure 1:**
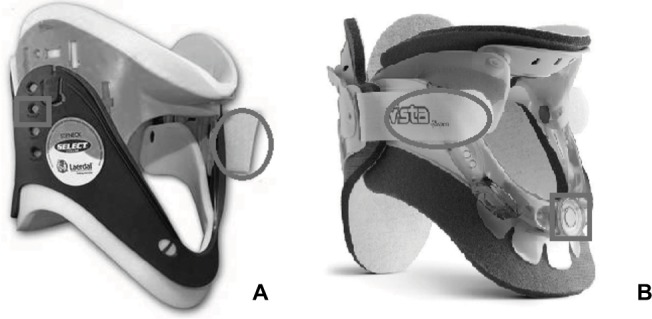
The StifNeck **(A)** and Aspen Vista **(B)** cervical collars. **Notes:** Tensioning straps are highlighted with gray circles, and height adjustment mechanisms with gray squares.

Two different C-collars were used for the present study (Figure 1), the Aspen Vista collar (Aspen Medical Products, Irvine, CA, USA) and the StifNeck (Laerdal Medical, Orpington, UK). Interface pressures were monitored using a commercial system (Mk III; Talley Medical, Romsey, UK), which incorporated individual 18 mm diameter cells with a reported mean error of 12±1% and repeatability of ±0.53 mmHg.^20^ Microclimate at the device–skin interface was measured using combined sensors (SHT75; Sensiron AG, Switzerland), recording relative humidity and temperature at a frequency of 0.5 Hz with a reported accuracy of ±1.8% RH and ±0.3°C, respectively. The physiological reaction of the skin was assessed by detecting a single inflammatory cytokine, IL-1α, present in sebum collected from the skin surface using Sebutape (CuDerm, Dallas, TX, USA), following a validated protocol.^21^ To determine the effectiveness of the collars, cervical spine range of motion (ROM) was measured using a handheld digital inclinometer (SOAR, Digital Level meter 1700). With the collar in situ, participants were also asked to report their perceived discomfort using an 11-point Numeric Rating Scale. The lowest score (0) represents no discomfort at any point, and the highest (10) represents extremely uncomfortable.

### Test protocol

Testing was performed in a biomechanics laboratory, with a controlled ambient temperature of 20°C±2°C and relative humidity of 40±5%. Participants were asked to have clean washed skin and have shaved where appropriate at least 48 hours before participation. Demographic data including, age, height, weight, and neck circumference were collected. Participants were then randomly fit with either the Aspen Vista collar (C1) or StifNeck collar (C2) in accordance with manufacturer instructions. Optimum fit (TO) was defined by a “secure fit” where a finger was restricted from sliding between the mandible and collar, without the chin protruding beyond the front piece. High (TH) and low (TL) fitting tensions were established by increasing and decreasing each strap tension by 5 mm and by increasing and decreasing collar height. Collar height was increased using a 90° turn of an adjustment dial on the Aspen collar. For the StifNeck design, a single incremental change using allocated notches was used above and below that of optimal. For each collar design, the height changes were ±15 mm from optimal. Once each tension was defined, the collar was removed prior to data collection.

**Figure 2:**
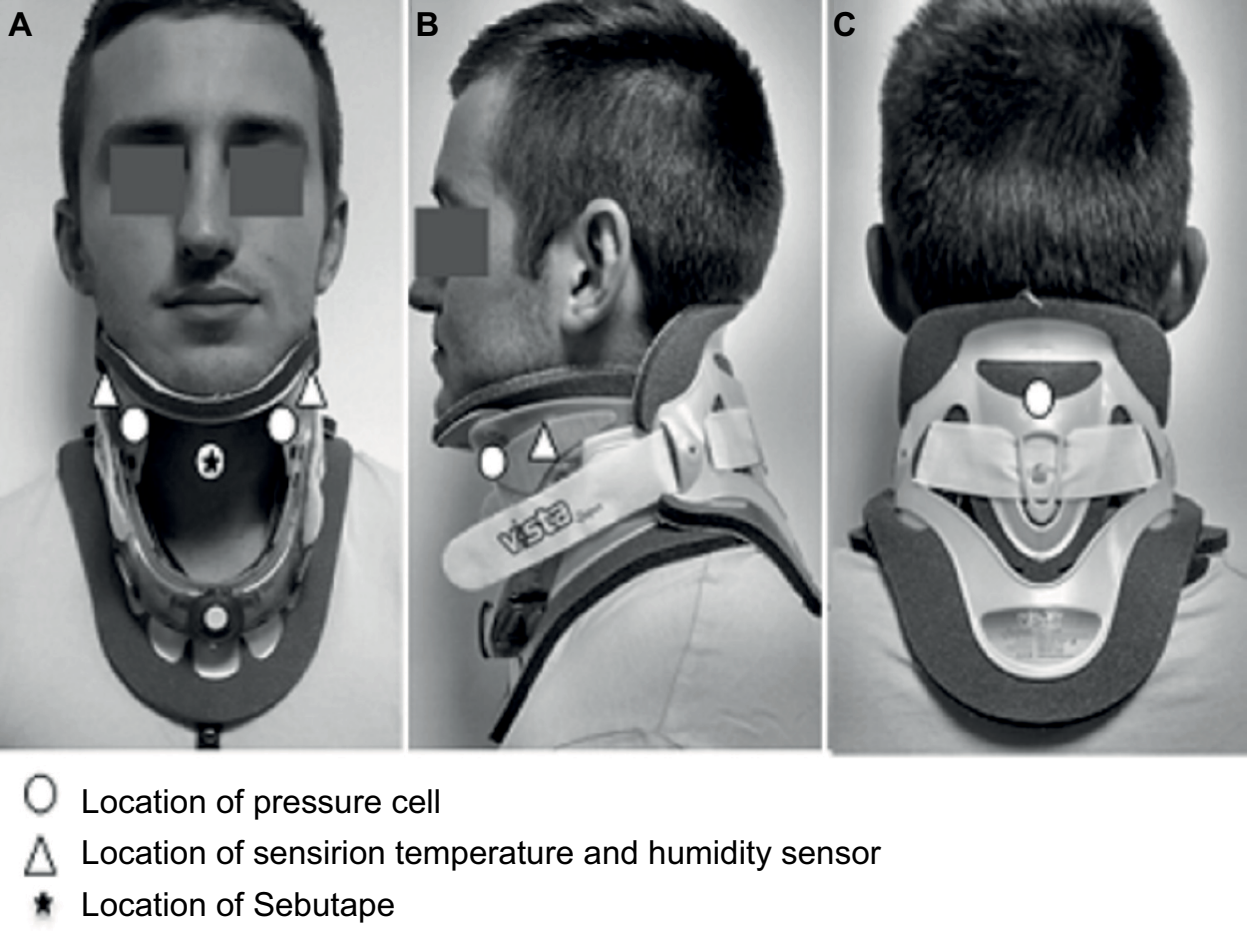
Measurement sites for interface pressure, temperature, humidity, and Sebutape recordings.

Initially, Sebutape samples were collected for a 2 minute period from the chin to provide unloaded baseline data. Following their removal, participants were fit with either the StifNeck or Aspen collar for 15 minutes at the three randomly applied tensions (TL, TO, and TH). Sebutape was then reapplied to the chin for the duration of collar application. With collar in situ, participants were asked to lay supine on a standard viscoelastic hospital mattress without pillow support. After 2 minutes, three interface pressures were recorded from the skin–device interface at the occiput, chin, and bilateral mandibular regions (Figure 2). Interface temperature and humidity measurements were recorded for one minute at the midway point of C-collar application. Participants were then asked to perform neck flexion, and right and left rotation, stopping when they experienced resistance from the collar. Flexion and rotation were measured with the inclinometer in the sagittal plane on the scalp and in the frontal plane on the forehead. One minute prior to collar removal, participants were asked to subjectively score their discomfort. To enable adequate soft tissue recovery, a 10-minute refractory period was imposed between each test condition. At the end of this period, another Sebutape was applied as a reference for the subsequent collar condition. Researchers regularly checked for skin blanching in accordance with NPUAP/EPUAP guidelines.^4^ All Sebutapes were removed and stored in coded vials at −80°C.

### Biomarker analysis

All Sebutape samples were prepared for analysis in accordance with a protocol described by Perkins et al.^21^ Samples were then analyzed using enzyme-linked immunosorbent assay kits to estimate IL-1α concentrations (Human IL-1α Standard ABTS ELISA Development Kit; PeproTech, Rocky Hill, NJ, USA). To account for differences in protein loading between each Sebutape, all absolute cytokine results were expressed as a ratio of post-:pre-collar application for each test condition.

### Data analysis

Statistical analysis was performed using IBM SPSS statistics V22 (IBM Corp, Armonk, NY, USA). Data from each of the test parameters were examined for normality using histogram plots and Shapiro–Wilk tests. Parametric statistics (mean ± standard deviation) were found to be appropriate for analysis of interface pressure, microclimate and cervical ROM measurements. Nonparametric statistics (median, interquartile range) were applied to cytokine ratios and comfort scores. Two-way repeated-measures analysis of variance and Fried-man tests were used to evaluate the effect of collar design and tension on the various test parameters. Where appropriate, sphericity of data was assessed using Mauchly’s test and significance accommodated for using the Greenhouse–Geisser correction. Pairwise comparisons between individual tensions were evaluated using Bonferroni-corrected *t*-tests and Wilcoxon signed-rank tests. For all outcomes, the statistical significance level was set to *p*≤0.05.

## Results

### Interface pressures

**Figure 3:**
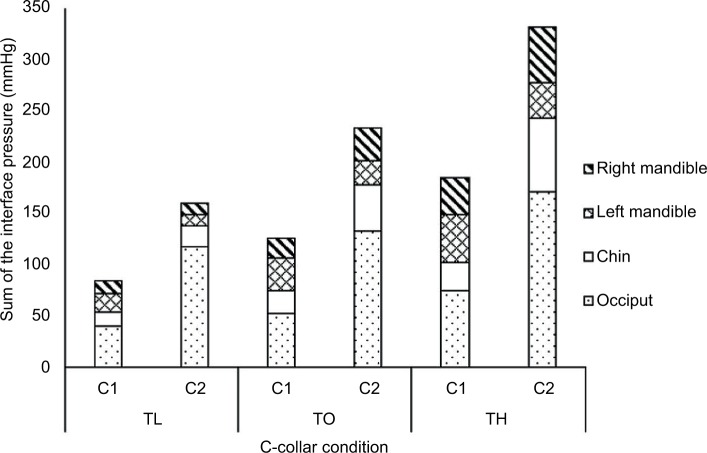
Stack plot of the interface pressure measurements at the device–skin interface during the application of Aspen (C1) and StifNeck (C2) designs, using the defined fitting tensions (low, optimal, and high). **Abbreviations:** C-collar, cervical collar; TL, low tension; TO, optimal tension; TH, high tension.

Table 1 illustrates the mean and standard deviation (SD) values of the interface pressures at each location for the three collar tensions and two collar designs. For each measurement location, there was a significant increase in interface pressures with greater collar tension (*p*<0.01, for both collar designs). The highest pressures observed from the selected measurement sites were at the occiput (Figure 3), which were significantly higher in each tension for the StifNeck compared to the Aspen collar design (*p*<0.05). In addition, there were some asymmetries between the left and right mandible values, notably, during the TO and TH test conditions for both collar designs.

On further examination of the data, there were no significant relationships (*p*>0.05) between interface pressures and either the BMI or neck circumference of the participants.

### Temperature and humidity

Table 1 also reveals that across different test conditions, there were no significant differences for either temperature or relative humidity values (*p*>0.05). Indeed, temperatures increased by 1.5°C from baseline unloaded values for all test conditions. However, with respect to the relative humidity changes, there were differences between the collar designs. Thus, the StifNeck design produced a 21% increase in relative humidity, compared to a 5.2% increase with the Aspen design.

### Cervical ROM

There were statistically significant differences in the cervical ROM for both flexion and total rotation between all three tensions (*p*<0.001) (Table 1). The mean ROM for flexion and rotation both decreased with an increase in collar tension. Greatest mean differences in ROM occurred between TO to TH tensions, with a mean decrease of 4° (38.5%) flexion and 14° (39.7%) total rotation. The StifNeck (C2) provided slightly more restriction across the three tensions, although these differences were not statistically significant (*p*>0.05).

### Effects of collar design and strap tension

The repeated-measures analyses revealed a significant effect of collar design on interface pressures at the occiput, right mandible, and chin (Table 2). In addition, collar design had a significant (*p*<0.001) effect on humidity, with the StifNeck having a greater increase than the Aspen collar. Irrespective of collar design, tension had the most significant effect across all the parameters, including all interface pressures, cervical ROM, and comfort. However, microclimate was not affected by collar tension.

### Cytokine analysis

**Figure 4:**
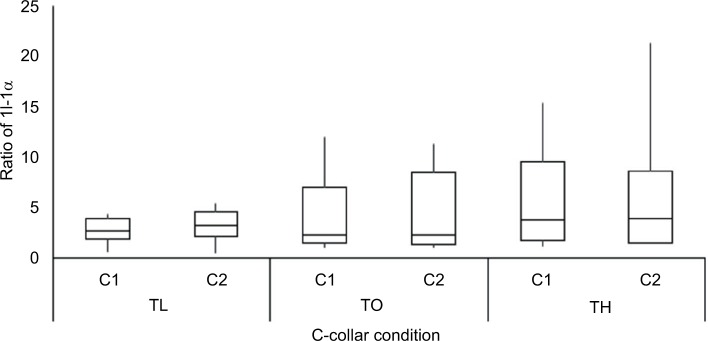
Ratio of the changes in inflammatory cytokine IL-1a from unloaded and loaded conditions using Aspen (C1) and StifNeck (C2) designs. **Notes:** Data have been collated for both collars across the three tensions. **Abbreviations:** C-collar, cervical collar; TL, low tension; TO, optimal tension; TH, high tension.

The ratio of cytokine concentrations from pre- to post-collar application revealed an increase (ratio >2) for all test conditions (Figure 4). Notably, the highest of these ratios were observed during the TH test condition for both collar designs, with a median ratio of 5.8 and 4.7 for the Aspen and StifNeck, respectively. Due to the variance in the data at both TO and TH conditions, there were no significant differences between either collar design or tension (*p*>0.05).

### Perceived comfort

There were statistically significant differences in subjective comfort scores between different collar fits (*p*<0.01), with mean discomfort greatest during the TH (Table 1). Although a trend was observed that the StifNeck design was more uncomfortable during TO and TH fitting conditions, the differences in the comfort scores between the two collar designs were not statistically significant (*p*>0.05).

## Discussion

The application of C-collars poses a significant risk for the development of MDRPUs.^3^,^5^ This study investigated the effects of varying C-collar design and fitting tension on the biomechanical, microenvironment, and biochemical response at the skin–medical device interface.^19^ The study revealed that increasing collar tension resulted in significantly higher interface pressures, decreased cervical ROM, and was associated with an increased subjective level of user discomfort. Collar design had a significant effect on the local microclimate between the device and the skin; in addition, both collars caused an upregulation of the proinflammatory biomarker, IL-1α, at loaded skin sites.

Interface pressures at all recorded locations increased with greater collar tension, with the highest recorded values at the bony prominence of the occiput. Interface pressure values were comparable to those previously reported in the supine position for the StifNeck (80±25 mmHg) and Philadelphia collars (57±25 mmHg),^13^ but considerably lower than those reported for Aspen Standard (119±49 mmHg) and Vista (122±48 mmHg) collars.^15^ These differences could have been caused by the selection of pressure mapping sensors (capacitive vs electropneumatic) or the professional expertise of the clinician fitting the collar (orthotist vs physiotherapist). However, all studies identified that the occiput represents a vulnerable bony prominence which is typically subjected to the highest loads. Consequently, the overlying skin tissues are at high risk of PUs.^22^ The large degree of variation in interface pressures observed between individuals is probably a result of the differences in face and neck morphologies. However, in contrast to previous studies,^15^ there were no associations between participants’ BMI or neck circumference and interface pressures. In addition, the asymmetries in some of the pressures (left and right mandible, Table 1) could have been due to face/neck morphology or the single-strap design of the collars creating asymmetries in collar alignment.

Detection of the proinflammatory cytokine IL-1α from skin sebum has previously been identified as a key indicator in loaded skin.^18^,^23^ However, this is the first study to investigate the physiological response of the skin to C-collar application. The results indicated an increase in IL-1α concentration in response to collar application, with median ratios of post-:pre-collar application between 2.4 and 5.8. The greatest ratio increase in IL-1α was recorded for the tightest collar application (TH). However, owing to variance in the response across the participants, no statistically significant differences were detected. This suggests that, relative to unloaded skin, collar application irrespective of design or fitting tension, led to an increase in this cytokine concentration. Previous investigation of the skin inflammatory response to varying tensions of a respiratory mask indicated ratio increase from basal levels of 1.2–1.3 for IL-1α.^19^ These differences in the magnitude of IL- α ratio could have been due to the anatomical site under investigation or the materials and application of the medical device. Recent research has also shown that prolonged lying on a spine board can increase IL1α and lactate, with both having a strong relationship with the period of lying down.^24^ However, more research is required to establish the time course of the inflammatory biomarker release following mechanical loading and the refractory period required for tissues to recover. In addition, altered levels of inflammatory mediators in plasma and urine may be associated with pressure ulcer development;^25^ further research is required to determine the optimal biomarkers for screening risk.

Irrespective of tension, collar application caused increased temperature and humidity at the skin–device interface relative to unloaded conditions. This was particularly evident with the StifNeck design made of stiff polymers materials and PVC foam inserts which limit the airflow at the device–skin interface. Clinical evidence^26^,^27^ and mathematical models^28^ both identify changes in microclimate as important factors in reducing skin tolerance to PUs. High humidity levels can induce softening of the stratum corneum^29^ with increased skin moisture contributing to a larger coefficient of friction,^30^ therefore increasing the likelihood of skin damage from shear forces. It was noteworthy that the two collar designs produced differences in humidity changes during application (Table 1). Thus, the Aspen collar with its modular design and use of foam materials, which allowed air flow, resulted in lower increases in humidity at the device–skin interface.

Current evidence suggests that posttrauma cervical immobilization is necessary to minimize the risk of further cervical or spinal cord injury.^31^ The present study revealed a statistically significant reduction in both cervical flexion and rotation ROM with increasing strap tension and collar height, a result which is similar to a previous investigation of collar height.^32^ ROM values recorded in the present study (Table 1) were higher than recently reported values of 4.7° for flexion and 35.9° for total rotation using an optimally fit Aspen Vista collar.^15^ These differences in ROM could have been due to participant bias, with individuals rotating and flexing their necks until they felt resistance. Despite multiple studies investigating the relative immobilizing efficacies of cervical orthoses,^33^,^34^ no guidelines specify the amount of restriction required for spinal protection. The results of the present study imply that there is a compromise between cervical restriction, the status of the skin tissue, and user comfort levels.

## Limitations

The present study is limited by its use of young healthy participants, which restricts the generalization of the findings particularly to hospitalized patients. Additionally, optimal collar application was based on the researchers’ interpretation of manufacturer guidelines and was not performed by a trained orthotist. Duration of collar application was also shorter than reported lengths of clinical application, although a physiological reaction at the skin was still evident (Figure 3).^35^ In addition, the time in which Sebutape was applied to the skin differed between baseline (2 minutes) and loaded conditions (15 minutes). However, this was applied consistently throughout the study providing the mean to assess relative changes in cytokine ratios between the test conditions.

Patients requiring C-collars may have reduced ability to sense and respond to noxious stimuli, including pressure, owing to reduced consciousness or neurological deficit.^5^,^11^ With the primary aim of restricting cervical ROM, coupled with poorly specified application guidelines, cervical collars are commonly fit incorrectly, thus increasing the risk of MDRPUs. This study highlights the impact of strap tension and collar design on perceived comfort and biomechanical and physiological outcomes, emphasizing the importance of correct fit to reduce the risk of PU development. Consequently, improved education for clinical staff regarding collar application and monitoring of MDRPUs should be considered to conform to current clinical guidelines,^4^,^36^ promoting regular skin and neurovascular assessments on patients with medical devices.^2^ Moreover, improved designs of collars which adapt to patient morphology and provide even pressure distribution and microclimate management could result in reducing collar-related PUs.

## Conclusion

This is the first study to specifically investigate the effects of C-collar design and fit on the physical and physiological response at the skin–device interface. The results revealed that increasing strap tension and collar height generated higher interface pressures at all contact sites, with the greatest values consistently recorded at the occiput. In addition, the material and design of the collars significantly affected both the pressure distribution and microclimate at the device–skin interface. Increased pressures were accompanied by significantly reduced cervical ROM and increased discomfort. Irrespective of tension, all collar applications consistently resulted in the release of the proinflammatory cytokine IL-1α at the skin surface. Therefore, collar application provides an environment conducive to PU development that can be influenced by collar design and fit.

## Figures and Tables

**Table 1:** Summary of parameters measured at the device–skin interface with the Apsen (C1) and Stifneck (C2) collar designs

Outcome measure	C1			C2		
	TL	TO	TH	TL	TO	TH
Interface pressure occiput (mmHg) mean ± SD	39±15	52±25^a^	72±34^b^,^c^	118±54	133±543^a^	171±56^b^,^c^
Interface pressure right mandible (mmHg) mean ± SD	13±7	19±10	35±15^b^,^c^	11±10	32±17^a^	53±26^b^,^c^
Interface pressure left mandible (mmHg) mean ± SD	19±8	33±14^a^	48±30^b^,^c^	12±10	24±18^a^	35±19^b^,^c^
Interface pressure chin (mmHg) mean ± SD	16±9	22±9	31±13^b^,^c^	20±12	45±28^a^	73±39^b^,^c^
ROM cervical flexion (°) mean ± SD	13±5	11±5	7±4^b^,^c^	14±8	9±4^a^	5±4^b^,^c^
ROM total cervical rotation (°) mean ± SD	47±19	34±12^a^	20±9^b^,^c^	56±20	36±11^a^	22±8^b^,^c^
Temperature change from baseline (°C) mean ± SD	1.6±1.0	1.5±0.6	1.4±0.7	1.1±0.8	1.3±1.0	0.6±1.1
Humidity change from baseline (% RH) mean ± SD	5.9±4.1	5.2±3.7	7.3±2.9	23±14	24±16	20±13
Comfort score (NRS/0–10) median (interquartile range)	3 (2–3)	3 (2–5)	6 (4–7)^b^,^c^	3 (2–4)	5 (3–6)	7 (4–8)^b^,^c^

**Notes:**

a Significant (*p*<0.05) difference between TL and TO conditions.

b Significant (*p*<0.05) difference between TL and TH conditions.

c Significant (*p*<0.05) difference between TO and TH conditions.

**Abbreviations:** TL, low tension; TO, optimal tension; TH, high tension; SD, standard deviation; ROM, range of motion; NRS, Numerical Rating Scale.

**Table 2:** Summary of the statistical analyses examining the effects of collar design (Apsen [C1] vs Stifneck [C2]) and strap tension

Outcome measure	C1 vs C2 *p*-value	Effect of tension *p*-value
Interface pressure occiput (mmHg) mean ± SD	<0.001	<0.001
Interface pressure right mandible (mmHg) mean ± SD	<0.05	<0.01
Interface pressure left mandible (mmHg) mean ± SD	0.4	<0.01
Interface pressure chin (mmHg) mean ± SD	<0.001	<0.001
ROM cervical flexion (°) mean ± SD	0.13	<0.001
ROM total cervical rotation (°) mean ± SD	0.06	<0.001
Temperature change from baseline (°C) mean ± SD	0.45	0.33
Humidity change from baseline (% RH) mean ± SD	<0.001	0.26
Comfort score (NRS/0–10) median (interquartile range)	0.45	<0.001

**Abbreviations:** SD, standard deviation; NRS, numerical rating scale; ROM, range of motion.
